# PD-1 Checkpoint Blockade in Patients for Acute Myeloid Leukemia after HSCT Relapse Resulted in Severe GVHD and sHLH

**DOI:** 10.1155/2022/1705905

**Published:** 2022-12-22

**Authors:** Zhi zhuo Du, Mi Zhou, Jing Ling, Lan Cao, Lingjun Kong, Shengqin Cheng, Peifang Xiao, Shaoyan Hu

**Affiliations:** Children's Hospital of Suzhou University, Suzhou, China

## Abstract

Treatment with immune checkpoint inhibitors (ICI) such as carrizumab leads to immune-mediated adverse effects including severe acute graft versus host disease (aGVHD) and secondary hemophagocytic syndrome (sHLH). Herein, we present two cases where aGVHD and sHLH developed after ICI administration, which was treated using methylprednisolone (MP). They developed high-grade fever complicated with liver dysfunction and diarrhea 1 day after ICI administration. Treatment with MP does not alleviate symptoms because of steroid resistance. Hyperbilirubinemia, rash with blisters, and watery diarrhea showed severe aGVHD. Hyperferritinemia, hypertriglyceridemia, and cytopenias suggested the diagnosis of HLH and met the criteria for sHLH diagnosis. They were thus administered intravenous high-dose MP, methotrexate (MTX), basiliximab, ruxolitinib, etc, which resolved these symptoms.

## 1. Introduction

With the progress of treatment, the therapeutic effect of acute myeloid leukemia (*AML*) has gradually increased from about 10% to about 70% [1], but allogeneic hematopoietic stem cell transplantation (*HSCT*) is the only treatment option for high-risk AML. AML recurrence after *HSCT* affects about 30–40% of *HSCT* recipients, and recurrence is still the primary cause of death in *AML* patients after *HSCT* [2]. The prognosis of *AML* recurrence after *HSCT* is poor. The 3-year survival rate is about 20–30% [3], and the treatment options are very limited. General management includes immunosuppressant withdrawal, chemotherapy, donor lymphocyte infusion (*DLI*), chimeric antigen receptor (CAR) T-cell therapy, and second transplantation, which are not very effective. At present, *ICI* is increasingly applied to solid tumors such as lymphoma, lung cancer, and melanoma, and has achieved encouraging therapeutic effects [4]. Therefore, increasing attention has been paid to the treatment of *ICI*. Some literature points out that highly expressed cells of programmed death 1 (*PD-1*) are closely related to leukemia relapse after *HSCT* [5–7], which provides a therapeutic target for leukemia recurrence after *HSCT*. However, *ICI,* including carrizumab, can activate T cells and induce serious autoimmune complications, such as immune-related adverse events (*irAE*), including *aGVHD*, secondary hemophagocytic lymphohistiocytosis (*sHLH*); *sHLH* is a potentially fatal disease, which can be manifested as fever, rash, cytopenia, coagulation dysfunction, liver dysfunction and hemophagocytic cells in the bone marrow. The occurrence of these two complications can endanger the lives of children.

Here, we report two *AML* patients who relapsed after HSCT treated with carrizumab, who developed *sHLH* and severe *aGVHD*, and were successfully treated with pulse therapy using high-dose *MP* and other immunosuppressants.

## 2. Case Report

### 2.1. Case 1

A 10-year-old girl was diagnosed with *AML (M4)*; chromosome: chromosome:46, *XX*; negative fusion gene; *Izkf1* negative; full exon sequencing: a frameshift mutation was detected in *WT1* gene; *RNA SEQ* detected after the fusion gene: *PRDM16/SKI* positive. The patient received induction chemotherapy with daunorubicin, cytarabine, cytarabine, and etoposide (*DAE*) scheme in June 2020 and achieved a partial response (*PR*). Then, the patient received consolidation chemotherapy with idarubicin, cytarabine (*IA*) scheme, and 2 cycles of dicitabine, homoharringtonine, cytarabine, and recombinant human granulocyte colony-stimulating factor (*G-CSF*) (*DEC* *+* *HAG*) scheme and achieved a PR. After receiving dicitabine, cladribine, cytarabine, and *G-CSF (DEC* *+ CLAG)* scheme, the patient achieved a complete response (*CR*); at this stage, the patient was positive for minimal residual disease (*MRD*), confirmed through flow cytometry (*FCM*). The patient underwent *allo-HSCT* from a *HLA*-mismatched related donor (5/10), after preconditioning with semustine, *CLAG*, cyclophosphamide, busulfan (*CCNU* *+* *CLAG* *+* *BuCy*), followed by cyclosporine A, mycophenolate mofetil, and short-term methotrexate for prophylaxis of *GVHD*. The patient achieved CR with *MRD* negativity (*CRMRD*-) 1 month after *allo-HSCT* and no *GVHD*. In May 2021, 4 months after *HSC*T, the disease progressed to relapse, and the evaluation of bone marrow (*BM*) showed that 16.5% of blasts, 84.6% of donor chimeric; 32.29% of *FCM–MRD*; and 7.57% of WT1. We performed the *HLA-loss* test, but no *HLA* gene loss was detected. So the patient was treated with azacytidine chemotherapy and *DLI*, but it was still not achieved *CR*. The second transplantation was performed 5 months after *HSCT1*. The patient underwent *allo-HSCT* from a HLA-mismatched unrelated donor (cord blood) (7/10), after preconditioning with total body irradiation (*TBI*), *CLAG*, mefallen, followed by cyclosporine A, mycophenolate mofetil, and short-term methotrexate for prophylaxis of *GVHD*. The patient achieved *CR MRD* 1-month after *allo-HSCT* and no *GVHD*. The patient was treated with azacytidine and interferon for preemptive therapy, but after that, the patient achieved *CR MRD*, but the copy number of *WT1* in the patient increased gradually. Three months after the *HSCT2*, the patient was treated with azacytidine and camrelizumab (3 mg/kg/d) injection. On that day, the child felt general pain and discomfort, low fever, gradually developed a skin rash, even blisters, diarrhea, dark green watery stool, jaundice, abnormal liver function, 4-degree *GVHD*, accompanied by repeated fever hyperferrinemia, hypofibrinogenemia, hypertriglyceridemia, cytopenias, and sIL-2R > 2500 pg/ml (Table 1), which was consistent with *sHLH*. The children were combined with four degrees of *GVHD* and *sHLH* and were gradually reduced and treated with methotrexate (*MTX*) and MP (5 mg/kg/d), and basiliximab. *GVH*D and *sHLH* were gradually controlled. The evaluation of bone marrow (*BM*) showed *CR*, no hemophagocyte was found, and the copy number of *WT1* decreased.

### 2.2. Case 2

A 2 years old girl was diagnosed with *AML (M7*); frameshift mutation was detected in the *ETV6* gene of myeloid line 67, and *CBFA2T3-GLS2* was positive. The patient received induction chemotherapy with daunorubicin, cytarabine, cytarabine, and homoharringtonine (*DAH*) scheme in March 2021 and achieved a *CR* with *CR MRD+*. After receiving *HAG*, 2 cycle *HA*, cytarabine, and etoposide (*EA*) scheme, the patient still achieved a *CR* with *CR MRD*; The patient underwent *allo-HSCT* from a *HLA*-mismatched unrelated donor (cord blood) (8/10), after preconditioning with *CCNU* *+* *CLAG* *+* *BuCy*, followed by cyclosporine A, mycophenolate mofetil, and short-term methotrexate for prophylaxis of *GVHD*. The patient achieved *CR MRD- 1* month after *allo-HSCT* and with 1-degree skin *GVHD*. In November 2021, 3 months after *HSCT*, the disease progressed to relapse, and the evaluation of bone marrow (*BM*) showed that 34% of blasts, 97.6% of donor chimeric; 46.29% of *FCM–MRD*; and 87.91% of *CBFA2T3-GLS2*; considering the relapse of *AML*, and camrelizumab (3 mg/kg/d) targeted antitumor therapy was given. Fever occurred on the same day and then gradually appeared the same 4-degree *GVHD* symptoms as the first case, such as a rash (including blisters), diarrhea, abnormal liver function, and jaundice. At the same time, the child developed repeated fever, hyperferrinemia, hypertriglyceridemia, cytopenias, *sIL-2R* >2500 pg/ml, and decreased *NK* cell activity (Table 1), which was consistent with sHLH. The child was complicated with four degrees of *GVHD* and *sHLH*, which were gradually reduced after being treated with *MTX* and *MP* (10 mg/kg/d), basiliximab, ruxolitinib, etc. *GVHD* and *sHLH* were gradually controlled. The evaluation of *BM* showed that 88% of blast parents gave up treatment.

## 3. Discussion

At present, chemotherapy, *DLI*, cell therapy, *et, al* are still unsatisfactory for patients with AML relapse after *HSCT*. *PD-1* pathway can be used as a checkpoint to limit T-cell-mediated immune response. Blocking the *PD-1* receptor on T cells can lead to the activation and proliferation of T cells, and induce an effective antitumor effect of immunotherapy [8]. In recent years, it has achieved certain therapeutic effects for patients with AML relapse after transplantation. Blocking *PD-1* after *HSCT* can enhance the graft *versus* leukemia (*GVL*) effect, but at the same time, the activation and proliferation of T cells can also lead to fatal *aGVHD* [9, 10]. Bradley et al. reported that the application of PD-1 inhibitors can also induce severe *aGVHD* [11]. In this *GVHD* state, T cells are activated and promote the activation of secondary macrophages, IL-6, IL-1, and IFN-*γ*, It is reported that *PD-1* inhibitors may cause *sHLH*, mainly in lymphoma, lung cancer, and other solid tumors [12, 13]. However, *PD-1* leads to fewer reports of post-transplant *HLH*. At present, the overall mortality of post-transplant *sHLH* is high, which can reach 80% [14]. If large doses of glucocorticoids cannot be rapidly alleviated, immune activation will continue, leading to further progress of *sHLH* and a*GVHD*, which will endanger life.

After the application of *PD-1*, the incidence rate of *irAE* is high. Both *aGVHD* and *sHLH* are *irAE*, but rarely occur at the same time. High fever, rash, diarrhea, and liver dysfunction were initially considered as *aGVHD* until there were cytopenia, decreased natural killer (*NK*) cell activity, hepatosplenomegaly, and coagulation dysfunction, especially hyperferrinemia (serum ferritin >10000 *μ*g/ml), which has important clinical significance for the diagnosis of *sHLH* [15], Will realize the occurrence of *sHLH*. For patients with these two complications at the same time, the treatment is often difficult. Single-use of glucocorticoids is considered effective for *irAE* [16, 17]. However, patients with *aGVHD* and *sHLH* may have glucocorticoid resistance, and other immunosuppressants such as basiliximab, *MTX*, and ruxolitinib need to be used to control the deterioration of *irAE*. Because of the high mortality rate of *sHLH* after transplantation, it is essential for early diagnosis and treatment of *sHLH*. For patients who have had *aGVHD*, if they have abnormal blood coagulation function, hyperferrinemia, splenomegaly, and other manifestations, they should be alert to the occurrence of *sHLH*.

Because the relapse of *AML* after *HSCT* is always a difficult point, the chemotherapy effect is poor and the overall survival rate is low. The treatment of *PD-1* inhibitors provides us with the possibility of treatment. In order to avoid the high incidence rate of *aGVHD* and *sHLH*, we may adopt the following methods: (1) whether to use *ICI* can be determined according to the values of *PD-1*, *PD-L1,* and *PD-L2*. Some studies point out that the proportion of *PD-1* or *PD-L1* is relatively high, which has a good therapeutic effect [18, 19]; (2) by reducing the therapeutic dose of *ICI,* the current conventional dose is 3 mg/kg, and the proportion of severe *aGVHD* or *sHLH* is relatively high, so the dose can be further reduced. Matthew et al. tried to reduce the dose to 1 mg/kg or even 0.5 mg/kg [20]. If children with *irAE* I or II can choose to receive *ICI* treatment again, or even increase the dose if it is level III or IV, it should be avoided; (3) at the same time as *ICI* treatment, we used glucocorticoid, cyclosporine, and other drugs to prevent *aGVHD* to reduce the incidence rate of *aGVHD* or *sHLH*. Once a*GVHD* occurs, we should be alert to *sHLH*. If *sHLH* occurs, the *HLH-94* regimen seems to achieve a better therapeutic effect.

## 4. Conclusion

In conclusion, these 2 cases represent a particularly early and aggressive form of *sHLH* with *PD-1* inhibitor therapy and report severe *aGVHD* caused by recurrent *PD-1* after *HSCT*. With the continuous exploration of new *PD-1* inhibitors in children with post-transplantation relapsed *AML*, it should be considered that the incidence rate of *sHLH* and *aGVHD* as possible severe *irAE* is increasing, and the methods and strategies for *ICI* treatment of post-transplantation relapsed *AML* can be improved, to achieve the best treatment effect and minimize toxic and side effects.

## Figures and Tables

**Table 1:** Frequency of acute treatment, emergent *GVHD,* and laboratory findings: pretreatment, day 14 after the administration of carrizumab.

	Case 1		Case 2	
	Pretreatment	Day 14	Pretreatment	Day 14
Stage of *aGVHD*				
Skin	Stage 0	Stage 4	Stage 1	Stage 4
GI	Stage 0	Stage 4	Stage 0	Stage 4
Liver	Stage 0	Stage 2	Stage 0	Stage 2

*HLH*				
Hemoglobin (g/L)	146	85	85	77
Platelet (×10^9^/L)	194	61	108	16
Neutrophil count (×10^9^/L)	1.64	1.58	1.64	0.03
Bilirubin (mg/dL)	0.88	4	0.22	4.11
Ferritin (ng/mL)	6253.6	20920	3796.7	10450
Fibrin (g/L)	2.81	0.71	2.85	4.94
Triglyceride (mmol/L)	1.76	4.94	2.56	5.35
sIL-2R (U/mL)	—	12365	—	76806
NK cell activity (<15.11%)	—	11.22	—	8.61

Acute GVHD stage/grade and responses were scored according to consensus and National Institutes of Health (NIH) criteria, GI = gastrointestinal.

## Data Availability

The raw data supporting the conclusions of this article will be made available by the authors, without undue reservation.
